# Hyperglycaemic hyperosmolar state and cerebral thrombophlebitis in paediatrics: A case report

**DOI:** 10.1002/edm2.389

**Published:** 2023-02-01

**Authors:** Maud Injeyan, Sabine Baron, Benjamin Lauzier, Benedicte Gaillard‐Le Roux, Manon Denis

**Affiliations:** ^1^ Department of Pediatrics CHU de Nantes Nantes France; ^2^ Department of Pediatric Endocrinology CHU de Nantes Nantes France; ^3^ Pediatric Intensive Care Unit CHU de Nantes Nantes France; ^4^ Université de Nantes, CHU Nantes, CNRS, INSERM, l'institut du thorax Nantes France

**Keywords:** case report, cerebral thrombophlebitis, diabetes, hyperglycaemic hyperosmolar state

## Abstract

**Introduction:**

Hyperglycaemic hyperosmolar state (HHS) is a known complication of type 2 diabetes mellitus; however, carbonated carbohydrate fluid intake may precipitate a more severe presentation of type 1 diabetes mellitus with hyperosmolar state. The management of these patients is not easy and can lead to severe complications such as cerebral venous thrombosis.

**Methods:**

We present the case of a 21‐month‐old boy admitted for consciousness disorders revealing a hyperglycaemic hyperosmolar state on a new‐onset type 1 diabetes and who developed cerebral venous thrombosis.

**Results and Conclusion:**

Emergency physicians should be aware of HHS in order to start the appropriate treatment as early as possible and to monitor the potential associated acute complications. This case highlights the importance of decreasing very gradually the osmolarity in order to avoid cerebral complications. Cerebral venous thrombosis in HHS paediatric patients is rarely described, and it is important to recognize that not all episodes of acute neurological deterioration in HHS or diabetic ketoacidosis are caused by cerebral oedema.

## Graphical abstract

Emergency physicians should be aware of HHS in order to start the appropriate treatment as soon as possible and to think about its acute complication. This case highlights the importance to restore euvolemia before insulin treatment and decrease very gradually the osmolarity in order to avoid cerebral complication. Cerebral venous thrombosis in HHS paediatric patients is rarely described, and it is important to recognize that not all episodes of acute neurological deterioration in HHS or DKA are caused by cerebral oedema.

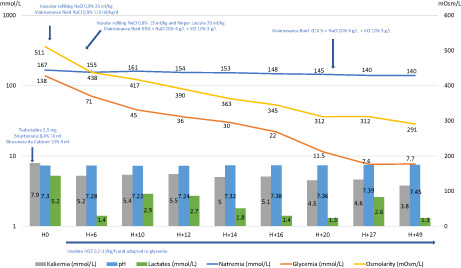

## 1 BACKGROUND

Diabetic ketoacidosis (DKA) and hyperglycaemic hyperosmolar state (HHS) are acute metabolic complications that occur in children with type 1 diabetes mellitus. Diabetic ketoacidosis is the most common hyperglycaemic crisis^1^ but HHS is associated with higher mortality^2^, ^3^, ^4^ and its incidence is increasing.^5^, ^6^, ^7^ HHS is a known complication of type 2 diabetes mellitus^8^; however, carbonated carbohydrate fluid intake may precipitate a more severe presentation of type 1 diabetes mellitus with hyperosmolar state.^4^, ^9^, ^10^ The main objective is to correct electrolyte disorders and avoid neurological complications. The common cerebral complication associated with HHS is cerebral oedema. While the risk of developing peripherical venous thromboembolism has already been described^11^, ^12^, ^13^ in DKA or HHS, cerebral venous thrombosis has been rarely described.^14^, ^15^ We present the case of a 21‐month‐old boy admitted for consciousness disorders revealing a hyperglycaemic hyperosmolar state for a new‐onset type 1 diabetes and who developed cerebral venous thrombophlebitis (CVT).

## 2 CASE PRESENTATION

A 21‐month‐old boy, weight 11 kg, presented at the emergency room with an unconscious and hypotonic state. He was born at 39 weeks of gestational age, with a birth weight of 3440 g and birth length of 51 cm. There was no family history of diabetes mellitus. He was previously healthy, presenting only a slight delay in psychomotor development as he could not sit up at 9 months nor stand alone at 21 months. He presented a rhinopharyngitis treated with amoxicillin one week before the hospitalization. He had a 3‐day history of polyuria and lethargy and a great alteration of the general condition for 2 days. His parents hydrated him only with sugar water. On the morning of the third day, he was found to be unresponsive in his bed.

Upon admission, he presented normal temperature (36.8°C), tachycardia (heart rate: 150 bpm), hypotension (blood pressure: 61/37 mm Hg), cold extremities, tachypnoea (respiratory rate: 40 cycles/min, SaO_2_: 100%) and dehydration signs objectified by dried mucous membranes. He presented unconscious (Glasgow coma scale [GCS]: 5) with no signs of localization and his pupils were in reactive mydriasis. Rapid glucose test was higher than 33 mmol/L. Blood tests reported hyperglycaemia (138 mmol/L), metabolic acidosis (pH 7.30, bicarbonate 16 mmol/L), hyperlactatemia (5.20 mmol/L), ketosis (0.9 mmol/L) and ketonuria. Electrolyte test revealed hypernatremia (sodium chloride 128 mmol/L and corrected sodium chloride 167 mmol/L) and hyperkalaemia (7.9 mmol/L). Creatinine and blood urea nitrogen were 159 μmol/L and 39 mmol/L, respectively, and serum osmolarity was 511 mOsm/L. Prothrombin rate and fibrinogen were 80% and 1.6 g/L, respectively. The electrocardiogram showed a regular sinus rhythm without any conduction disorder but hyperkalaemia signs including very large and sharp T waves and a diffuse ST shift.

An injected brain computed tomography scan (CT‐scan) was performed and showed a permeable veinous sinus and a hypodensity of the white matter in front of the temporal horn and the right ventricular carrefour. The patient received a volume expansion of 250 ml of isotonic fluid NaCl 0.9% followed by a rehydration with 110 ml/kg/day of NaCl 0.9%. Hyperkalaemia was treated by calcium gluconate 10% (4 ml), Terburtaline aerosol (1 aerosol of 2.5 mg), and sodium bicarbonate 8.4% (10 ml). His mental status required intubation under sedation by 2 mg/kg of ketamine and 1 mg/kg of rocuronium. He was then referred to the paediatric intensive care unit (PICU) and regular insulin (Insulin analogue [Lispro] 0.02 UI/kg/h) was started.

**FIGURE 1:**
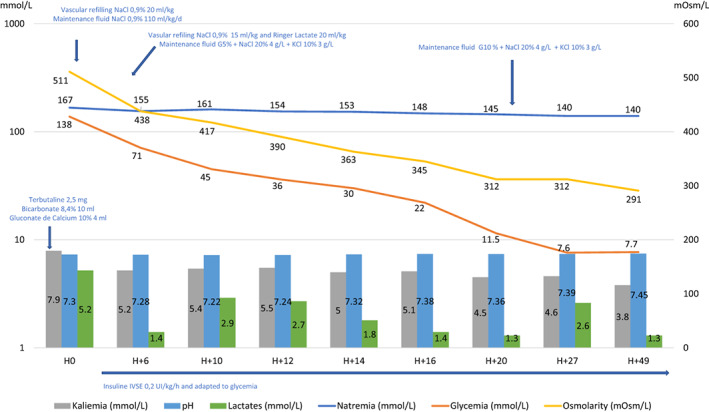
Changes in biological parameters and medical interventions over time upon admission. H, Hours

**FIGURE 2:**
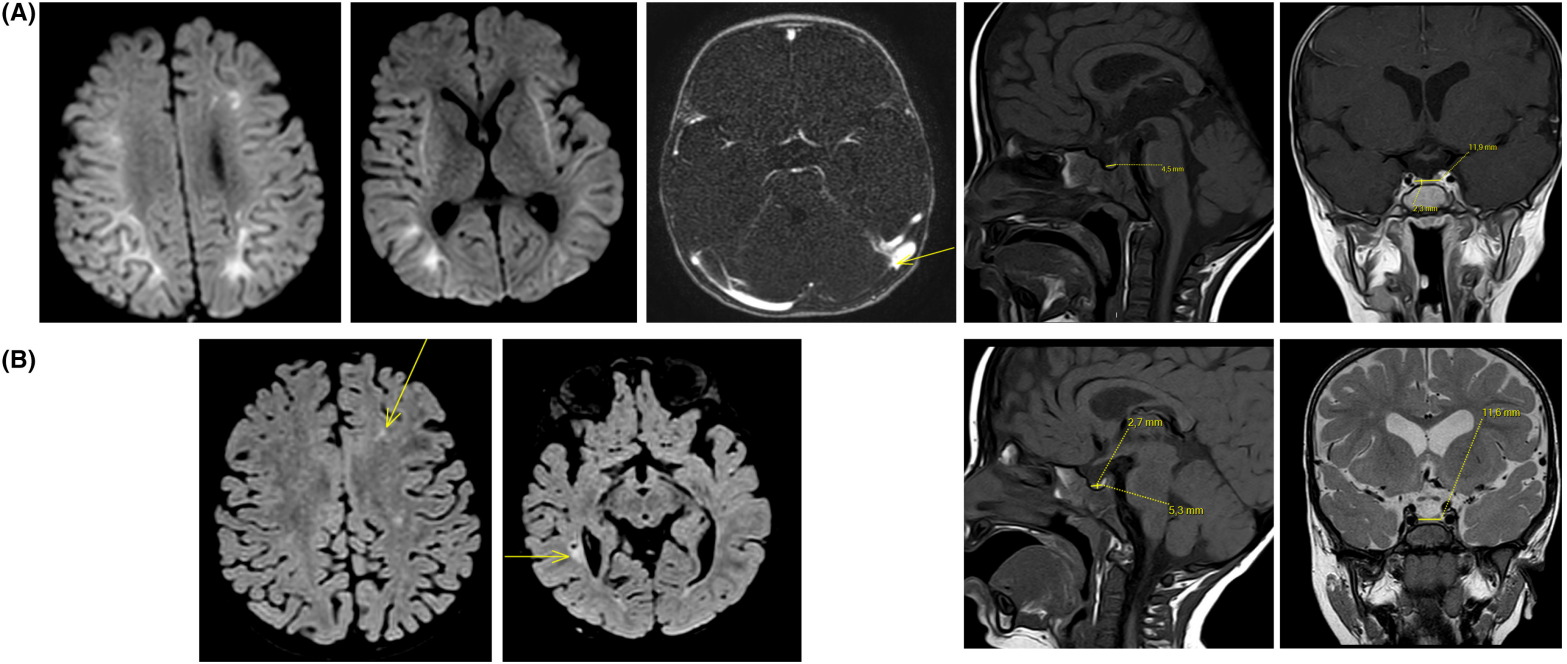
Magnetic resonance imaging performed on Day 2 and at 3 months. (A) MRI performed on Day 2, extensive thrombophlebitis of the superior sagittal sinus and of several cortical veins in the vertex, the torcular, the left transverse sinus and presence of small thrombi in the right transverse sinus. Significant bilateral and symmetrical signal abnormalities of the fronto‐parieto‐temporo‐occipital subcortical white matter and of the external capsules were also visible in diffusion restriction, which seemed rather to be related to metabolic disorders. (B) MRI at 3 months, partial permeabilization of the superior sagittal sinus with persistence of a heterogeneous aspect of the sinus, seat of some linear images probably corresponding to residual filiform thrombi, a complete permeabilization of the transverse sinuses and the torcular and cortical veins, and an appearance of FLAIR signal abnormalities of the periventricular white matter, probably sequelae of the abnormalities visualized in diffusion on the first MRI.

During PICU hospitalization, volume expansion was continued intravenous by 150 ml of NaCl 0.9% and 50 ml of ringer lactate and maintain intravenous fluid was changed for Glucose 5% with NaCl 20% 4 g/L and KCl 10% 3 g/L. Laboratory examinations were performed in order to check and modulate electrolyte changes (Figure 1). The serum sodium concentration and the serum osmolarity increased to a maximum of 167 mmol/L (corrected natremia) and to 511 mOsmol/L, respectively. Serum glucose level decreased and normalized to 7.6 mmol/L in 24 h, which led to a gradual decreased in serum osmolarity and sodium. This corresponded to an average drop of 1 mmol/L/h of sodium in the first 24 h, with a maximum of 3.5 mmol/L/h between H10 and H12, and of 7.37 mOsm/L/h of osmolarity with a maximum of 13.5 mOsm/L/h between H10 and H14. His hemodynamic status remained stable, without any vasopressor support requirement. The echocardiography showed normal cardiac function. The transcranial Doppler (TCD) performed did not show any anomalies. The patient was sedated by morphine and midazolam which was stopped on Day 2. At the initial examination without sedation and before extubation, the child presented a rolling of the lower and upper limbs, a pyramidal syndrome including bilateral Babinsky's sign, sharp reflexes with increased reflexogenic area and a withdrawal reaction to pain. Seizures were detected on Day 2 and treated by clonazepam (0.05 mg/kg) and then levetiracetam (40 mg/kg/day). No clinical signs of intracranial hypertension (ICH) were found and TCD was normal (pulsatility index (PI): 0.9 and diastolic velocity (Vd): between 30 and 40 cm/s). Electroencephalogram showed a marked encephalopathy pattern without epileptic activity, possibly of metabolic origin and related to sedation. His brain magnetic resonance imaging (MRI) reported an extensive thrombophlebitis of the superior sagittal sinus and of several cortical veins in the vertex, the torcular, the left transverse sinus as well as presence of small thrombi in the right transverse sinus. Significant bilateral and symmetrical signal abnormalities of the fronto‐parieto‐temporo‐occipital subcortical white matter and of the external capsules were also visible in diffusion restriction, which seemed rather to be related to metabolic disorders (Figure 2). He was immediately treated by anticoagulant (unfractionated heparin, 20 UI/kg/h, target antiXa: 0.3–0.5). The mental status improved and extubation was possible on Day 3.

Five days after PICU admission, the neurological examination showed no focal signs, symmetrical osteo‐tendon reflexes, no Babinski's sign, no peripheral hypertonia, no epileptoid tremor and some upper limb stereotypies. The brain CT‐Scan showed an absence of haemorrhagic remodelling and a stability of the extent of the thrombophlebitis reaching the superior sagittal sinus, torcular, left transverse sinus and some cortical veins. The white matter signal abnormalities described on the MRI were not visible on the CT scan. A Doppler ultrasound of the lower limbs showed a deep venous thrombosis in superficial femoral vein, to the location of the central catheter. Serology revealed positive antibody against insulin (4 unit/ml; normal high <0.4 unit/ml) and no antibody against GAD, IA2, ICA, Zn8. A central hypothyroidism was found (T4 3.7 pg/ml [9.4–18 pg/ml], TSH 0.46 μUI/ml [0.2–4 μUI/ml]). We did not find any abnormality on the other pituitary axes except a low IGF1 level (27 ng/ml [49–297 ng/ml]), controlled at 38 ng/ml two days later, which may be related to dehydration and acute malnutrition.

The infant was transferred to the general paediatric ward on Day 5 and discharged home on Day 26. Initially, he was treated with a regimen of basal and rapid insulin and then transitioned to an insulin pump. L‐Thyroxine treatment was stopped at 1 month with normalization of the thyroid balance (TSH 0.21 μUI/ml; T4 10.6 pg/ml); without any antibodies stating a transient acute central hypothyroidism; and diabetes was well balanced. Chromosomal analysis by CGH‐Array was normal and the search for fragile X syndrome was negative. The metabolic workup was unremarkable (mucopolysaccharides, urinary oligosaccharides, plasma lysosphingolipids and Gaucher disease). In addition, the mitochondrial DNA study was normal. He was treated with anticoagulants (antivitamin K: coumadin 2 mg per day) for 3 months. The brain MRI performed at 3 months showed partial repermeabilization of the superior sagittal sinus with persistence of a heterogeneous aspect of the sinus, seat of some linear images probably corresponding to residual filiform thrombi, a complete repermeabilization of the transverse sinuses and the torcular and cortical veins, and an appearance of FLAIR signal abnormalities of the periventricular white matter, probably sequelae of the abnormalities visualized in diffusion on the first MRI (Figure 2). The neurological examination returned to baseline, and he is followed up in a specialized neurodevelopment centre.

## 3 DISCUSSION

We report the first case of HHS revealing a new‐onset type 1 diabetes complicated by cerebral thrombophlebitis in a young boy. In France, diabetes mellitus incidence increases by 4% each year since 1988.^16^ HHS remained a rare diagnosis in paediatrics but hyperosmolar events had higher rates of complications.^4^ In this case, the little boy was thirsty, and his parents gave him only sugar water. That probably contributed to an increase in blood sugar and the hyperosmolar state. Several case reports have shown that carbonated carbohydrate fluid intake may precipitate a more severe presentation of type 1 diabetes mellitus in adolescents,^10^ but this presentation is rarely described in children. The mortality rate in patient with HHS varies between 20% and 60% and timely diagnosis as appropriate treatment prevent complications and death.^2^, ^3^, ^17^

The child presented with hyperglycaemia without acidosis, which could have led to a delayed diagnosis of diabetes 1. Contrary to diabetic ketoacidosis, HHS is characterized by development of a severe hyperglycaemia without acidosis, these patients maintain a low insulin production leading to the absence or underproduction of ketones via lipolysis. The pathophysiology of HHS is further compounded by a disordered renal response. HHS commonly occurs after polyuria and polydipsia, resulting in profound dehydration. This is accompanied by severe electrolyte imbalance, greater than in DKA because of the longer duration of osmotic diuresis. The hypertonicity of the hyperosmolar state preserves intravascular volume, which contributes to masking clinical signs of dehydration which can lead to a difficult and delayed diagnosis.^7^, ^18^

HHS is a life‐threatening emergency. The treatment of HHS requires rehydration, electrolyte balance, intravenous insulin and management of complication. The patient received a volume expansion of 250 ml of isotonic fluid NaCl 0.9% followed by a rehydration with 110 ml/kg/day of NaCl 0.9% and then insulin after his transfer in PICU. The priority is to restore euvolemia before the use of insulin. Fluid replacement should be started with 0.9% saline in order to maintain circulatory volume, restore renal perfusion and gradually correct hyperosmolarity.^19^ It is commonly admitted that hypotonic maintenance intravenous fluids lead to a greater risk of developing cerebral oedema in children, and thus its use should be avoided.^3^ We monitored for signs of ICH through clinical examination and DTC during the first 48 h. Adequate fluid replacement must begin before insulin administration for many reasons. First, early insulin is unnecessary in HHS, because ketosis is usually minimal.^20^ Secondly, insulin bolus could induce shifts of potassium to the intracellular space and hypokalaemia^20^; move intravascular water into the cells, then exacerbating hypotension and increasing the risk of brain oedema.^19^ Furthermore, fluid administration alone promotes dilution and decreases serum glucose. Finally, the osmotic pressure exerted by glucose may fall rapidly then leading to circulatory compromise and venous thrombosis.^20^

HHS can cause many complications. The main risks of severe dehydration and hyperviscosity are brain oedema, rhabdomyolysis, pancreatitis, renal failure, malignant hyperthermia and thromboembolism. This state can lead to death with refractory arrhythmia or multisystem failure (cardiac, pulmonary oedema and renal failure).^21^ The severity of altered mental status correlates with the level of hyperosmolarity.^3^ In our case, the patient presented a severe dehydration with hyperosmolarity and hyperviscosity that altered mental status. The brain MRI and doppler ultrasound showed an extensive cerebral thrombophlebitis and thrombosis in superficial femoral vein, respectively. Adult literature reported that patients with diabetes and hyperosmolarity had a significantly higher risk of venous thromboembolism (VTE).^11^ Several studies showed that young children (<3 years old) with DKA have an increased incidence of clinical VTE associated with the placement of femoral central venous catheters (CVC)^12^, ^13^ and these patients had significantly higher glucose and corrected sodium concentrations.^13^ Cerebral venous thrombosis in the course of diabetic hyperglycaemia seems to be extremely rare. Only two reports were described in the literature. In the first one, venous thrombosis was interpreted as a consequence of the combination of dehydration and iron deficiency anaemia but no hyperosmolarity was reported.^14^ The second one reported an 8‐year‐old male patient with CVT at early stage of management and was associated with hyperosmolarity.^15^ In the context of cerebral thrombophlebitis, anticoagulation is effective in reducing the risk of death, sequelae in acute phase and of recurrence.^22^ There is no recommendation for prophylactic anticoagulation for patients with HHS. Anticoagulation treatment should be reserved for children who require CVC and are immobile for more than 24 to 48 hours.^23^

## 4 CONCLUSION

HHS is an increasingly common complication of T1D but its diagnostic and management remains difficult. Emergency physicians should be aware of HHS in order to start the appropriate treatment as soon as possible and to think about its acute complication. This case highlights the importance to restore euvolemia before insulin treatment and decrease very gradually the osmolarity in order to avoid cerebral complication. Cerebral venous thrombosis in HHS paediatric patients are rarely described, and it is important to recognize that not all episodes of acute neurological deterioration in HHS or DKA are caused by cerebral oedema. Thromboprophylaxis is not recommended but early anticoagulation in case of cerebral thrombophlebitis may improve prognosis.

## AUTHOR CONTRIBUTIONS

**Maud Injeyan:** Conceptualization (equal); data curation (equal); investigation (equal); resources (equal); writing – original draft (equal). **Sabine Baron:** Writing – review and editing (supporting). **Benjamin Lauzier:** review and editing (supporting); validation (supporting). **Benedicte Gaillard‐Le Roux:** Data curation (supporting); resources (supporting); writing – original draft (supporting); writing – review and editing (supporting). **Manon Denis:** Conceptualization (equal); data curation (equal); supervision (lead); validation (lead); writing – original draft (lead); writing – review and editing (lead).

## CONFLICT OF INTEREST

The authors declare that they have no competing interest to disclose.

## CONSENT FOR PUBLICATION

Written informed consent was obtained from the patient's legal guardians for publication of this case report and any accompanying images. A copy of the written consent is available for review by the Editor‐in‐Chief of this journal.

## Data Availability

All data supporting the findings of this article are included in the article.
